# Magnetic field responses in *Drosophila*

**DOI:** 10.1038/s41586-024-07320-4

**Published:** 2024-05-01

**Authors:** Charalambos P. Kyriacou

**Affiliations:** Neurogenetics Group, Department of Genetics & Genome Biology, University of Leicester, Leicester, UK

**Keywords:** Molecular neuroscience, Theoretical chemistry

arising from: M. Bassetto et al. *Nature* 10.1038/s41586-023-06397-7 (2023)

Bassetto et al.^1^ reported that *Drosophila* are unable to detect magnetic fields using a conditioning^2^ and negative geotaxis assay^3^, and on this basis, they dismiss these and all further experimental studies published on *Drosophila* magnetic fields^4–12^. Critically, fly magnetic geotactic responses were replicated independently by Bae et al.^12^, yet this important and extensive confirmatory study is not discussed. Furthermore, Bae et al. successfully demonstrated a magnetic field conditioning response^12^, underlining how experienced *Drosophila* groups can successfully negotiate magnetic paradigms. I have reanalysed the data from all three geotactic experiments from Bassetto et al.^1^ and, despite serious flaws in methodology, their results reveal that *Drosophila* detect magnetic fields.

In the geotaxis experiments of Fedele et al.^3^, the percentage of male flies climbing 15 cm in 15 s generated the maximum separation between sham responses to blue light (BL) and those to red light (RL), which provide the critical positive controls. Forty-eight per cent of CS-LE males exposed to BL reached this criterion, compared to 12% in RL (that is, about 36% absolute, 400% relative enhancement in BL; Fig. 1a). The contention of Bassetto et al.^1^ that flies should fall into either climber or non-climber categories and not reflect an underlying Gaussian distribution does not stand serious scrutiny. Bae et al.^12^ carried out similar experiments, comparing geotaxis at about 0 μT magnetic field in darkness (approximately equivalent to RL for flies) and white light (500 lx, including BL). They observe a geotactic difference of about 300% between the two lighting conditions expressed as positive geotaxis (non-climbers; Fig. 1b). Experiment 1 of Bassetto et al.^1^ replicates the procedure of Fedele et al.^3^ in equipment I provided, but apparently using mixed groups of males and females. It is of concern that geotactic responses are barely different between the sham BL and RL critical positive controls (Fig. 1a). I recalculated that the CS-LE strain reached 26% criterion under BL with 22% under RL, whereas corresponding values for the more active CS-OX were 52% (BL) and 45% (RL) (Fig. 1a). Given these tiny absolute and relative differences between RL and BL, compared to those in previous studies^3,12^, one questions how any magnetic field effect could be detected in such limited phenotypic space. Evidently, Bassetto et al.^1^ did not suspect a problem with these positive controls (see Supplementary Information for the probable reason). In addition, strain CS-LE is considerably less active in BL than in Fedele et al.^3^, possibly owing to inbreeding, as I originally provided a single vial of this line. Consequently, I predominantly limit my reanalyses to CS-OX, which is as active in BL as CS-LE is in Fedele et al.^3^ (Fig. 1a).

**Fig. 1:**
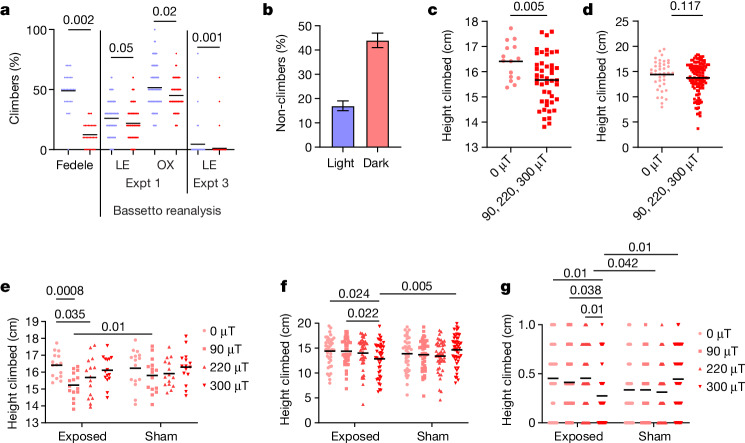
Results of reanalysis of geotaxis data in Bassetto et al.^1^. Raw data are shown for all experiments; horizontal black lines represent means. **a**, Reanalysis of Bassetto et al.^1^ raw data for positive control conditions. The plot shows a comparison of raw data for climbing response of CS-LE flies under sham to RL and BL (red and blue, respectively) conditions from Fedele et al.^3^ with raw data for CS-LE and CS-OX (experiment (Expt) 1) reanalysed from Bassetto et al.^1^. The *y* axis shows the percentage of flies that reached 15 cm in 15 s. Mean climbing scores averaged over 10 trials for each tube for the experiment of Fedele et al.^3^ (one-tailed *t*_4_ = 5.82, *P* = 0.002, based on 3 replications each for RL and BL; total observations, *n* = 60). Experiment 1 of Bassetto et al.^1^ has 300 observations under sham, divided equally between BL and RL, in which 60 tubes (each with 10 flies) are tested 5 times. The raw data and mean responses are shown for CS-LE and CS-OX. For ANOVA, the proportion of flies reaching criterion is calculated for each tube (strain: *F*_1,117_ = 17.07, *P* « 0.0001; light: *F*_1,117_ =9.82, *P* = 0.002; interaction: *F* = _1,117_ 0.09, not significant (NS); based on *n* = 121 average climbing scores from 5 trials (total flies, *n* = 601)). False discovery post hoc values are shown (see Supplementary Information). In experiment 3 of Bassetto et al.^1^, only 26/208 (12.5%) and 6/199 (3%) CS-LE trials produced flies that reached criterion in BL and RL, respectively, so 182 and 193 trials, respectively, had a score of 0. The mean percentage of five trials in which individual flies reached criterion and Mann–Whitney *U*-test result comparing BL to RL are shown. It is clear from the raw data that there is barely any overlap between RL and BL climbing scores in the positive controls of Fedele et al.^3^, whereas the overlap is considerable in the raw data for experiments of Bassetto et al.^1^. **b**, Results of the experiment of Bae et al.^12^ comparing climbing in darkness and white light at 0 μT. Redrawn from Bae et al.^12^; raw data not available. Data are mean ± s.e.m. **c**, Reanalysis of climbing of 0-μT-exposed CS-OX flies in groups of 10 individuals compared to higher 90-, 220- and 300-μT exposures from gravity experiment 2 of Bassetto et al.^1^ (one-tailed *t*_58_ = 2.64, *P* = 0.005, *n* = 60). **d**, Same analysis and comparison for Flyvac experiment 3 of Bassetto et al.^1^, in which individual CS-OX flies are tracked (one-tailed *t*_160_ = 1.19, *P* = 0.117, *n* = 162). **e**, Reanalysis of gravity experiment 2 with CS-OX from Bassetto et al.^1^. Mean height (horizontal bar) climbed per tube in 15 s. ANOVA, exposure versus sham: *F*_1,112_ = 1.42, NS; exposure intensity: *F*_3,112_ = 4.67, *P* = 0.004; interaction: *F*_3,112_ = 0.8, NS; *n* = 120. False discovery post hoc *P* values shown. **f**, Reanalysis of Flyvac experiment 3 with CS-OX from Bassetto et al.^1^. Mean height climbed per tube in 15 s. Exposure versus sham: *F*_1,333_ ≈ 0, NS; exposure intensity: *F*_3,333_ = 0.38, NS; interaction: *F*_3,333_ = 3.44, *P* = 0.017; *n* = 341; false discovery post hoc *P* values shown. **g**, Proportion of flies that reached criterion of 15 cm in 15 s from Flyvac experiment 3. The horizontal bar depicts the mean. ANOVA, exposure versus sham: *F*_1,348_ = 1.47, NS; exposure intensity: *F*_3,348_ = 0.19, NS; interaction: *F*_3,348_ = 4.40, *P* = 0.0036, *b* = 356; false discovery post hoc *P* values shown. **b**, Redrawn from ref. ^12^, Creative Commons Attribution 4.0 International License (http://creativecommons.org/licenses/by/4.0/).

In experiment 2, Bassetto et al.^1^ expose groups of 10 individuals to 0 μT, at which Earth’s magnetic field is neutralized, compared to 90-, 220- and 300-μT exposures with corresponding sham (ambient, about 40-μT) controls. They do not use 500-μT exposures as in Fedele et al.^3^. Inspecting the automated tracking for CS-OX revealed 1,062,956 frames logged from an expected 1,800,000 (accuracy 59%). Importantly, no positive controls were carried out involving RL versus BL for CS-OX. Nevertheless, taking their results at face value, the prediction^3,12^ is that flies should climb higher at 0 μT compared to magnetic field exposure. Reanalysis of their data reveals significantly higher climbing at 0 μT than at 90-, 220- and 300-μT exposures combined (Fig. 1c). Also predicted is that 0-μT-exposed flies should climb higher than corresponding shams, but the higher-intensity exposures should reduce climbing compared to sham, generating an interaction. Figure 1e reveals that at 90-, 220- and 300-μT exposures, climbing is reduced compared to corresponding shams, as expected (but not significantly), whereas there is little difference between 0 μT compared to its sham.

For Flyvac experiment 3, Bassetto et al.^1^ tracked individual flies. The accuracy of the tracking is 84.9%, considerably better than experiment 2. In CS-LE, 12.5% (26/208) of BL trials included flies that reached the climbing criterion (15 cm in 15 s in at least 1 of 5 trials), compared to 3% in RL (6/199). The mean percentage of flies across all trials reaching criteria was 4% for BL and 1% for RL, so this criterion cannot be used to investigate magnetic field effects (Fig. 1a). Nevertheless, I detected significant differences between the lighting conditions using Fisher exact (*P* = 0.004) and Mann–Whitney (*P* = 0.001) tests, reflecting absolute BL-to-RL enhancement of 9.5%, relative 415%. Consequently, I recalculated the mean height climbed at 15 s for CS-LE under sham in RL and BL, which was 6.17 cm to 8.75 cm (142% BL enhancement), considerably better than experiment 1. Yet again, positive RL and BL controls were not carried out for CS-OX, so I assumed that CS-OX discriminates BL and RL as well as CS-LE does. I therefore took the average height climbed for CS-OX individuals at 15 s and reanalysed the data. The prediction is that 0-μT-exposed flies should climb higher than those of the other exposures combined. The prediction is partially fulfilled, but unlike experiment 2, the difference is not significant (Fig. 1d). Flies exposed to 0 μT should also climb higher in BL than sham (about 40 μT), but at 300 μT, sham flies should climb higher than exposed flies. Two-way analysis of variance (ANOVA) reveals a significant interaction generated by the flies at 0 μT climbing higher than sham, with a strong reciprocal significant response at 300 μT (Fig. 1f), so the prediction is fulfilled. A similar result is obtained when I examined the percentage of CS-OX flies reaching criterion (15 cm in 15 s), noting how much higher CS-OX climb than CS-LE in BL (compare experiment 3 in Fig. 1a with Fig. 1g). One wonders what the result would have been had Bassetto et al.^1^ used an exposure of 500 μT (as in Fedele et al.^3^), as the magnetic field effect in this particular single-fly paradigm seems to gain momentum with increasing intensity. The over-elaborate and highly conservative ANOVA of Bassetto et al.^1^ (see Methods of ref. ^1^) produced non-significant results, after which the authors did not seem to interrogate their data further. Had they inspected carefully the relevant part of their own figures (see my Supplementary Fig. 1), they might have thought twice about their conclusions.

I have shown that the positive controls for experiment 1 worked poorly, if at all, and that in experiment 2, comparing 0-μT exposures to the higher exposures gave the expected result, despite poor tracking accuracy and no positive controls. In the more robust final experiment, despite no positive controls, the interaction expected, in which flies climb higher under 0 μT and lower under higher exposures compared to sham, also gave the predicted result. Instead of engaging in some relatively simple troubleshooting for each paradigm, increasing BL intensity in experiment 1 (and perhaps experiments 2 and 3), and tuning up the tracking software in experiment 2, Bassetto et al.^1^ preferred the option of simply racking up large (108,609) numbers. It is extraordinary that no positive RL or BL controls were carried out for CS-OX, because it has long been known that fly strains differ in their responses to RL^13^.

Finally, one wonders why Bassetto et al.^1^ dismissed all fly magnetic field experiments^2–12^ from eight independent groups using different paradigms. Bassetto et al.^1^ state that because flies do not use a navigational compass, they have no use of a magnetic sense. They ignore the demonstration of Bae at al.^12^ that flies use the Earth’s magnetic field to fly low. *Drosophila melanogaster* feed and oviposit on decaying fruits that lie mainly at ground level, so a magnetic sense would be adaptive for foraging. In turn, this suggests that magnetoreception is primary, and the functions it serves, foraging or navigation, lie downstream. Furthermore, magnetic field effects can be mediated in flies by the 52-residue cryptochrome (Cry) carboxyl terminus alone without the canonical FAD-binding site and the 3–4 Trp residues required to generate radical pairs in Cry, results obtained using adult circadian behaviour (under impeccably controlled conditions) and single-larval-motoneuron physiological assays^8,10,11^. Mouritsen, Hore and collaborators favour a model in which full-length avian CRY4 with FAD binding and Trp tetrads is required for detecting magnetic fields, based on in vitro spectroscopy experiments on CRY4 peptides circumstantially allied to behavioural evidence from bird navigation studies^14^. Clearly the two competing hypotheses, Cry C terminus versus full-length Cry, although not mutually exclusive, are at odds. The critically flawed attempt of Bassetto et al.^1^ to cast doubt on all fly magnetic field work, together with their statement that (genetically and molecularly inscrutable) night-migratory songbirds are the best organism for understanding the underlying mechanism of light-dependent magnetoreception (ignoring the molecularly tractable navigating monarch butterfly^15^), should be seen clearly in this context.

## Reporting summary

Further information on research design is available in the Nature Portfolio Reporting Summary linked to this article.

### Supplementary information

Supplementary Information Supplementary text regarding experiments 1–3 and Supplementary Fig. 1.

Reporting Summary

## Data Availability

All of the raw data for Bassetto et al.^1^ on which the analyses are based can be found at Open Science Framework (10.17605/OSF.IO/HZ98Q).
