# Supplementary material for: Magnetic field responses in Drosophila
Source: Nature. 2024 May 1;629(8010):E3–5. doi: 10.1038/s41586-024-07320-4 (PMC11062905; doi:10.1038/s41586-024-07320-4)

nature portfolio

https://doi.org/10.1038/s41586-024-07320-4

Supplementary information

Magnetic field responses in *Drosophila*

In the format provided by the
authors and unedited

Supplementary Information

All the data from Bassetto et al were publicly available and downloaded from their OSF submission. Re-analysis was performed using excel spreadsheets, pivot tables and Graph Pad Prism software.

Experiment 1. Replication of Fedele et al[3]

Each tube was given a single score based on the average number of flies to reach criterion over 5 repeat trials in RL/BL. As flies (presumably males and females) were in groups of 10 per tube, the maximum number of flies that could reach criterion for each tube was 50, so the proportion of flies attaining criterion was used as the dependent variable.

When the equipment was returned to me after the Oldenburg experiments, the maximum BL output that could be obtained was 0.07μW cm^{-2} and not the 0.25μW cm^{-2} used in Fedele et al. The problem was defective smoothing capacitors which had failed (they swell over time with use). They had been 'frozen' the last time they were used in Oldenburg, so I suspect that the intensity of BL was much reduced for most of their experimentation and would have been difficult to pick up with the human eye or a camera that automatically adjusts brightness. This is the most parsimonious explanation for the very poor RL v BL responses. No doubt Bassetto et will claim they monitored the BL intensity continually, but they do not report this for experiment 1. Irrespective of the cause, their behavioural data (Fig 1A) reveal that the BL was not sufficiently intense enough to enhance climbing compared to RL.

'Gravity' experiment 2.

I initially attempted to analyse the raw data at 15s but the data was very sporadic for the 5 trials (59% of possible frames logged) so I calculated the average climbing position between 14 and 15s which provided sufficient data and (almost always) 5 trials for each tube. I collapsed against blocks (3 tubes in each block) unlike Bassetto et al who use Blocks as a separate variable in their ANOVAS. Blocks has no biological meaning and is simply used as another variable to test against Main effects and interactions, leading to an extremely conservative analysis. Benjamini & Hochberg test for false discovery rate was used for *posthoc* comparisons. Bassetto et did not perform any positive RL v BL controls for CS-OX.

Flyvac Experiment 3

Positive RL/BL controls were only performed for CS-LE. Raw data were analysed as in experiment 2 but each trial reports the height climbed by a single fly. The average height reached by each fly over the 5 trials was used unless there was only data for a single trial. Even when I included the 8 flies that were excluded in this manner it did not change the ANOVA results (Interaction p= 0.021). I was also able to use the data to calculate the proportion of CS-OX flies that reached criterion height of 15 cm in 15s (Fig 1F). Again, positive RL v BL controls were not performed for the CS-OX strain.

Fig S1. Results of Bassetto et al's Flyvac experiment 3 showing the 0μT and 30μT exposures and corresponding shams for CS-LE (left panel) and CS-OX) right panel. Not how in both strains the 0μT exposed flies climb higher than sham but this is reversed at 300μT as predicted[3,4]. None of these effects were significant by Bassetto et al's ANOVAs. Redrawn from Bassetto et al Supplementary figures Fig S13b and Fig S15

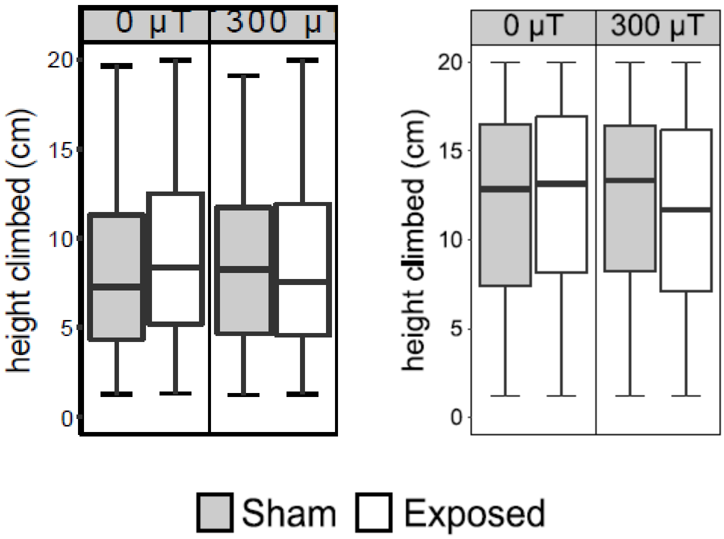

Supplement: Supplementary file 1 — Supplementary text regarding experiments 1–3 and Supplementary Fig. 1. [file 41586_2024_7320_MOESM1_ESM.pdf]
