# Supplementary material for: Magnetic field responses in Drosophila
Source: Nature. 2024 May 1;629(8010):E3–5. doi: 10.1038/s41586-024-07320-4 (PMC11062905; doi:10.1038/s41586-024-07320-4)
Supplement: Supplementary file 2 — Reporting Summary [file 41586_2024_7320_MOESM2_ESM.pdf]

nature portfolio

Corresponding author(s): C. P. Kyriacou

Last updated by author(s): Feb 29, 2024

Reporting Summary

Nature Portfolio wishes to improve the reproducibility of the work that we publish. This form provides structure for consistency and transparency in reporting. For further information on Nature Portfolio policies, see our Editorial Policies and the Editorial Policy Checklist.

Statistics

For all statistical analyses, confirm that the following items are present in the figure legend, table legend, main text, or Methods section.

n/a	Confirmed	
☐	☒	The exact sample size (*n*) for each experimental group/condition, given as a discrete number and unit of measurement
☐	☒	A statement on whether measurements were taken from distinct samples or whether the same sample was measured repeatedly
☐	☒	The statistical test(s) used AND whether they are one- or two-sided<br>*Only common tests should be described solely by name; describe more complex techniques in the Methods section.*
☒	☐	A description of all covariates tested
☐	☒	A description of any assumptions or corrections, such as tests of normality and adjustment for multiple comparisons
☐	☒	A full description of the statistical parameters including central tendency (e.g. means) or other basic estimates (e.g. regression coefficient) AND variation (e.g. standard deviation) or associated estimates of uncertainty (e.g. confidence intervals)
☐	☒	For null hypothesis testing, the test statistic (e.g. *F*, *t*, *r*) with confidence intervals, effect sizes, degrees of freedom and *P* value noted<br>*Give P values as exact values whenever suitable.*
☒	☐	For Bayesian analysis, information on the choice of priors and Markov chain Monte Carlo settings
☒	☐	For hierarchical and complex designs, identification of the appropriate level for tests and full reporting of outcomes
☒	☐	Estimates of effect sizes (e.g. Cohen's *d*, Pearson's *r*), indicating how they were calculated

Our web collection on statistics for biologists contains articles on many of the points above.

Software and code

Policy information about availability of computer code

Data collection	data collected by Bassetto et al https://doi.org/10.17605/OSF.IO/HZ98Q
Data analysis	GraphPad Prism

For manuscripts utilizing custom algorithms or software that are central to the research but not yet described in published literature, software must be made available to editors and reviewers. We strongly encourage code deposition in a community repository (e.g. GitHub). See the Nature Portfolio guidelines for submitting code & software for further information.

Data

Policy information about availability of data

All manuscripts must include a data availability statement. This statement should provide the following information, where applicable:

- Accession codes, unique identifiers, or web links for publicly available datasets
- A description of any restrictions on data availability
- For clinical datasets or third party data, please ensure that the statement adheres to our policy

Data availability. All the Bassetto et al raw data on which the analyses are based can be found at https://doi.org/10.17605/OSF.IO/HZ98Q

Human research participants

Policy information about studies involving human research participants and Sex and Gender in Research.

Reporting on sex and gender	Bassetto et al are unclear about the sex of flies used. Only in the gravity geotactic experiment do they specifically state that male flies were used. In the Bassetto PhD thesis he states that for the third experiment (FlyVac) 'the same gender was used' as in the gravity experiment so I assume they used males too. In experiment 1, the direct replication of Fedele et al, they do not state anything about gender, so I presume male and female flies were used. This is unfrrtunate as male and female flies will interact in the climbing assay,thereby adding another source of uncontrolled variation.
Population characteristics	n/a
Recruitment	n/a
Ethics oversight	n/a

Note that full information on the approval of the study protocol must also be provided in the manuscript.

Field-specific reporting

Please select the one below that is the best fit for your research. If you are not sure, read the appropriate sections before making your selection.

☒ Life sciences ☐ Behavioural & social sciences ☐ Ecological, evolutionary & environmental sciences

For a reference copy of the document with all sections, see nature.com/documents/nr-reporting-summary-flat.pdf

Life sciences study design

All studies must disclose on these points even when the disclosure is negative.

Sample size	All the reanlayses are based on the large sample sizes of Bassetto et al. The degrees of freeedom and sample sizes used in the statistical tests are provided
Data exclusions	For Flyvac experiment 3 Bassetto took 5 trials for each individual male. However 8 males generated data for only 1 trial so an average climbing score across trials could not be computed. Nevertheless, the data was analyses both with and without these 8 datapoints, but the statistical results were the same (see Supplementary Methods)
Replication	This study represents a re-analysis of data by Bassetto et al who could not replicate the results of Fedele et al (2014). The re-analysis reveals that in fact the Bassetto et al data support the conclusions of Fedele et al, so the initial non-replication by Bassetto et al appears to be flawed.
Randomization	Basssetto et al randomised the order of sham and exposure
Blinding	Bassetto et al apparently performed their experiments blind

Reporting for specific materials, systems and methods

We require information from authors about some types of materials, experimental systems and methods used in many studies. Here, indicate whether each material, system or method listed is relevant to your study. If you are not sure if a list item applies to your research, read the appropriate section before selecting a response.

Materials & experimental systems

n/a	Involved in the study	
☒	☐	Antibodies
☒	☐	Eukaryotic cell lines
☒	☐	Palaeontology and archaeology
☐	☒	Animals and other organisms
☒	☐	Clinical data
☒	☐	Dual use research of concern

Methods

n/a	Involved in the study	
☒	☐	ChIP-seq
☒	☐	Flow cytometry
☒	☐	MRI-based neuroimaging

Animals and other research organisms

Policy information about studies involving animals; ARRIVE guidelines recommended for reporting animal research, and Sex and Gender in Research

Laboratory animals	Drosophila melanogaster
Wild animals	n/a
Reporting on sex	Only one geotactic experiment out of three reports the sex used. Another probably used males (FlyVac experiment 3), but no mention is made for the sex of that animals in experiment one, the direct replication of Fedele et al
Field-collected samples	n/a
Ethics oversight	none required for Drosophila

Note that full information on the approval of the study protocol must also be provided in the manuscript.
